# The Dental Caries Pandemic and Disparities Problem

**DOI:** 10.1186/1472-6831-6-S1-S2

**Published:** 2006-06-15

**Authors:** BL Edelstein

**Affiliations:** 1 Section of Social and Behavioral Sciences, College of Dental Medicine, Columbia University, New York, NY, USA; Childrens Dental Health Project, Washington DC, USA

## Abstract

Understanding caries etiology and distribution is central to understanding potential opportunities for and likely impact of new biotechnologies and biomaterials to reduce the caries burden worldwide. This review asserts the appropriateness of characterizing caries as a "pandemic" and considers static and temporal trend reports of worldwide caries distribution. Oral health disparities within and between countries are related to sugar consumption, fluoride usage, dental care, and social determinants of health. Findings of international and U.S. studies are considered in promoting World Health Organization's and others' recommendations for science-based preventive and disease management interventions at the individual, clinical, public health, and public policy levels.

## Introduction

### "Pandemic"

Understanding caries etiology and distribution is central to understanding the potential opportunities for and likely impact of new biotechnologies and biomaterials to reduce the caries burden worldwide [1]. The term *pandemic *is customarily reserved for global disease outbreaks that are acute and fatal, such as the influenza epidemic of 1918 that killed tens of millions globally. In contrast to the term *epidemic*, from the Greek language roots for "upon" and "the people," pandemic refers to a disease that is visited upon "all the people." It suggests an impact on populations of entire countries, continents, or much of the world. The term therefore implies two elements: global distribution and severe consequence. By characterizing dental caries as a pandemic, symposium organizers have focused attention on caries as a highly prevalent disease around the globe. They have also implied that it has profound individual and societal significance because of its often severe, though non-fatal, consequences.

Caries is both diet-dependent and fluoride-mediated and is amenable to prevention and management at both the individual and population levels. It is also readily treatable through conventional surgical interventions and dental repair. Therefore, the extent and severity of its consequence for individuals, communities, and nations varies by the availability and balance of these factors. As a result, there are marked disparities in caries experience, treatment experience, and disease consequences both between countries and within countries. The term *pandemic *is fitting because those who are affected by caries and have little or no access to care number in the hundreds of millions, reside on all continents and in most societies, and experience significant consequences of pain and dysfunction that impair their most basic functions of eating, sleeping, speaking, being productive and enjoying general health as defined by the World Health Organisation.

### Static View of Caries Distribution

**Figure 1:**
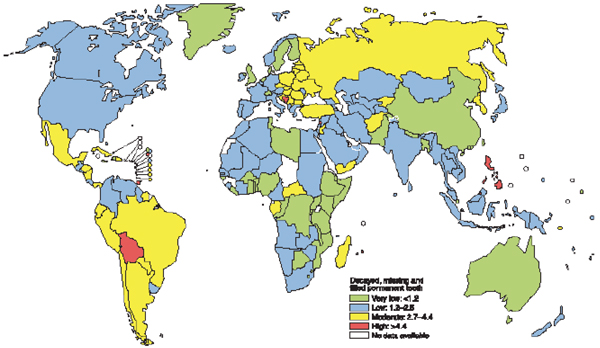
Dental caries levels (DMFT) of 12-year-olds worldwide. World Health Organization [2].

The World Health Organization's 2003 report on oral health [2] provides an overview of global caries epidemiology that confirms its international pandemic distribution. Globally, WHO reports caries prevalence in school-age children at 60–90% and as virtually universal among adults in the majority of countries [3]. Because so few countries are spared high levels of this disease, caries maps typically display disease severity rather than prevalence. Figure 1 displays caries distribution among 12 year olds by average numbers of teeth affected, using the Decayed, Missing, and Filled Teeth (DMFT) index of severity. The map shows a clear pattern of higher disease experience in North and South America, Western Europe, and much of Africa; more moderate disease experience in much of South America, Russia, and the former Soviet Republics; and low levels of disease in Eastern Africa, China, Australia, and Greenland. While the correlation between caries rates and national development is not tight, WHO has observed that developed countries have higher rates of caries experience, while developing countries have lower rates [2]. WHO has attributed these differences to the relative availability of simple sugars in diets, to fluoride, and to dental treatment. U.S. findings by the Centers for Disease Control and Prevention (CDC) [4] released in August 2005 reveal high ongoing prevalence of dental caries in children, with 27% of preschoolers, 42% of school-age children, and 91% of dentate adults having caries experience. Paralleling international findings of country [5,6] and family level [7,8] income-related disparities (dubbed "dental caries polarization") [9], the new U.S. report reveals ongoing [10] marked disparities by income. For example, primary tooth caries prevalence is 1.8 times greater for children of poverty than for those with incomes twice the poverty level.

### Dynamic View of Caries Distribution

Konig [11] notes that the "[caries] situation worldwide was and remains today extremely variable and changes are occurring in different directions." Commenting on these variances, Petersen *et al*. [3] note that the "current pattern of oral disease reflects distinct risk profiles across countries related to living conditions, lifestyles and environmental factors, and the implementation of preventive oral health schemes." These factors typically relate to differences in disease experience across countries but also reflect social gradients within countries. The study of social determinants of health when applied to oral health suggest that stages of societal development as well as individual circumstances play critical roles in caries acquisition and expression.

International correlates of pediatric caries experience have been explored in a search for appropriate nation-level preventive policies and programs. Comparing disease rates in 109 countries, higher levels of childhood caries were found to correlate with total sugar consumption, urbanization, and level of development but not with gross domestic product, total healthcare spending, or dentist-to-population ratio [12]. The quality of care provided to individuals suffering the affects of dental caries, however, does vary by provider availability as extraction is more common than dental repair in countries with fewer dentists [3].

Longitudinal global trends described in the WHO report [2] demonstrate the "tyranny of the mean" when considering the dynamism of caries' distribution in populations. Average caries rates worldwide among 12-year-olds expressed as DMFT have remained reasonably steady, around 2.5, since 1980. However, trend lines for developed and developing countries are diametrically different. Developed countries have experienced almost linear decreases in caries rates among the benchmark 12-year-olds from >4.5 DMFT in 1980 toward the mean, while developing countries have experienced ongoing increments also approximating the mean.

While caries is declining in some countries and increasing in others, once established in a population, it does not decline to pre- sugar availability baseline levels except under extraordinary circumstances of deprivation, [13,14]. As sugar accounts for the majority of variance in caries rates among countries [15] and continues to be readily available once introduced, the majority of polled international cariology experts concur that changes in sugar consumption contribute considerably less to caries declines relative to the contribution of fluorides [16].

CDC's cross-sectional trend analysis over a recent decade [4] parallels WHO's findings for developed countries, with caries declining significantly (7.4%) in permanent teeth of school-age children and adolescents. However, a trend toward increased primary tooth caries and a growing subpopulation of Latino children with higher caries rates suggests that the next cohort of U.S. children may demonstrate a reversal in caries declines.

### Disparities and Social Determinants of Dental Caries

Increasing attention is being paid to differences in population subgroups in characterizing the distribution and correlates of dental disease, particularly in children [17-20]. Petersen and Lennon [21] summarize these differences, stating that "Despite great improvements in the oral health of populations across the world, problems still persist particularly among poor and disadvantaged groups in both developed and developing countries." Even within a single country, disparities by social standing exist in large part because of differences in diet, fluoride use, and social empowerment. Disparities by social empowerment persist both because of lack of access to dental care [22] and despite such access [23], since differences in care utilization vary even when care is available.

Theoretical frameworks that explicate pathways between social, behavioral, and political factors and health [24] are being applied to oral conditions [25] to identify relationships that may be actionable. These factors have been identified as strong correlates, if not determinants, of oral health in populations and sub-populations [26] and have been considered as potentially fruitful factors for intervention to improve both oral and general health. WHO's recently formed Commission on Social Determinants of Health, like national health plans in England, Canada, and Sweden seek political interventions that hold promise to improve health by addressing such social determinants.

Concise messages to public policymakers regarding caries interventions have been developed by a number of organizations to promote public adoption of science- and technology-based interventions that hold strong promise to reduce the caries pandemic. For example, the Washington DC-based Children's Dental Health Project (, accessed September 5, 2005) states "Too many children suffer too much from a disease that is well understood and almost completely preventable. Childhood tooth decay is the rare example of a very common and consequential health problem that can be solved through public interventions without incurring extreme costs."

### Implications of Caries Epidemiology for Research, Clinical Practice, and Public Policy

"One-size-fits-all" approaches to prevention and disease management of individuals and groups fail profoundly to reflect important differences in disease experience within and between populations. Needed today are bio-technologic/bio-material, clinical, behavioral, and social interventions that are risk-based, subpopulation-targeted, age-specific, biologically sound, and safe and accepted at both individual and population levels. Typical approaches to dental care – whether limited to extractions or involving complex dental restorations – often fail to capitalize on current scientific understanding of disease distribution, correlates, and pathogenesis. By not thinking about, managing, and treating dental caries as the dynamic, progressive, infectious, diet-dependent, behavioral disease that it is, clinicians and program managers miss opportunities to bring the power of sound science to bear on a disease that remains prevalent and consequential to the daily lives of millions of children worldwide. This is due in part to the failure of technology- and knowledge-transfer from the cariology laboratory to the dental chair and family home so that providers and individuals can successfully shift their orientation from treating signs and symptoms of caries to managing the underlying disease process. Although caries is a disease that manifests throughout the lifespan, prioritizing children is appropriate because caries is first established in early childhood and plays out across the lifetime. Current conceptual frameworks that need to be addressed include shifting from characterizing dental caries as a condition to a disease; from passive to active management; from static to dynamic understanding of pathogenesis; from treatment to management; and from dento-centricity to individual and family centricity.

WHO recommends oral health interventions that (1) reduce disease burden through a "risk-factor" approach that focuses on high needs individuals and groups; (2) promote healthy lifestyles and reduce risk factors arising from environmental, economic, social, and behavioral sources; (3) develop oral health systems that equitably improve oral health outcomes, respond to legitimate needs, and are financially fair, and (4) integrate oral health into national and community health programs and promote oral health in public policy. The 2001 U.S. Surgeon General's invitational Workshop on Children and Oral Health focused attention on public and private policy interventions suitable for young children, including (1) start early and involve all who come in contact with young children and their families; (2) assure competencies of all providers; (3) be accountable through tracking and performance measures; (4) take public action through coalitions; (5) maximize utility of sound science; (6) improve public programs for the underserved; (7) grow an adequate and competent dental workforce; and (8) empower families to address their oral health [27].

## Conclusion

Noting the wide variation among countries in physical and economic resources available for dental care, WHO's Poul Erik [3] calls for prioritizing cost-effective preventive interventions over curative care. The biotechnological and biomaterials approaches to caries prevention and management proposed by this NIDCR funded Symposium (Biotech and Biomaterials Research to Reduce the Caries Epidemic) represent an essential and timely response to that call.

## Competing interests

The author(s) declare that they have no competing interests.
